# Exploring Neurocognitive Deterioration in Alzheimer’s Disease

**DOI:** 10.3390/biology12030343

**Published:** 2023-02-21

**Authors:** Ferdinando Franzoni, Davide Maria Cammisuli, Giorgia Scarfò, Gianluca Castelnuovo

**Affiliations:** 1 Department of Clinical and Experimental Medicine, University of Pisa, 20123 Milan, Italy; 2 Department of Psychology, Catholic University, 20123 Milan, Italy; 3 Psychology Research Laboratory, IRCCS Istituto Auxologico Italiano, 20123 Milan, Italy

Oxidative stress (OS) contributes to cerebral biochemical impairment and is defined as the imbalance between reactive oxygen species (ROS) and the antioxidant potential of neuronal cells pertaining brain activity. OS is implicated in several neurological conditions such as atherosclerosis, mild cognitive impairment (MCI), Parkinson’s disease, Huntington’s disease, amyotrophic lateral sclerosis, vascular dementia, and Alzheimer’s disease (AD) [1]. The central nervous system (CNS) is highly susceptible to reactive oxygen species (ROS) because of its richness in fatty acids that are sensible to peroxidation as well as a high oxygen consumption that exposes the brain to free-radical accumulation [2]. The overproduction of reactive species along with a failure in the balance of antioxidant systems results in the destruction of cellular structures, lipids, proteins, DNA, and RNA [3,4].

AD is characterized by a progressing cognitive impairment affecting several domains, with memory loss as the main feature. It represents the main cause of dementia, accounting for an estimated 60% to 80% of all cases [5], and is the most expensive and burdening disease in terms of sanitary costs and caregivers’ distress, respectively. AD is mainly characterized by the presence of beta-amyloid protein plaques and neurofibrillary tangles of hyperphosphorylated tau protein. Both of these proteins begin to accumulate in the brain several years before the manifestation of clinical symptoms. Nowadays, a more comprehensive understanding of the cellular preclinical phase of AD is claimed to understand the biological underpinnings of the disease. The prodromal phase of AD involves the clinical entity of mild cognitive impairment (MCI) or mild neurocognitive disorder (MND), as classified in psychiatry nosography [6]. This entity refers to the transitional state between normal aging and dementia and it consists of an abnormal cognitive function in one or more domains [Petersen, 2016] without loss of global independence in the activities of daily living. The term of “MCI *due to* AD” [7] is further used to describe a phase of slight cognitive impairment prior to overt dementia, with the presence of AD-related biomarkers. The biological definition of AD refers to the operationalization formulated by Jack and coll. [8] as the A(amyloid)/T(tau)/ neurodegeneration(N) biomarkers schema.

Antioxidant activity in the AD brain is reduced in comparison to age-matched controls [9]. An abnormal level of OS has been reported also in the blood stream of patients with AD and MCI with elevated markers of lipid peroxidation, a dysregulation of the copper metabolism, and a decreased antioxidant capacity [10]. Amyloid-beta peptide (Aβ) has been observed to trigger both OS and neuroinflammation [11,12]. Neuroinflammation is recognized as a relevant component of AD pathology, involved in disease progression and neurodegeneration [13]. Neuroinflammatory processes produce enhanced ROS and reactive nitric oxide species (RNS) reporting neurotoxic effects [14]. The gut microbiota dysbiosis is also linked to neuroinflammation in AD. The latest findings suggested that sodium oligomannate remodels gut microbiota and reduce peripheral inflammatory cell populations contributing to central inflammation [15]. Gut inflammation has been recognized as a possible cofactor mediating cognitive impairment in neurodegenerative disorders [16] and a sectorial meta-analysis showed that probiotics may confer mental health benefits to the host probably because of anti-inflammatory and anti-oxidative properties both in MCI and AD [17], even if more reliable evidence is needed to ascertain this.

AD pathology is also associated with mitochondrial dysfunction, specifically implicated in the brain energy metabolism and ROS generation through oxidative phosphorylation [18]. Mitochondria are one of the principal sources of intracellular ROS because of the aerobic metabolism and ATP synthesis [19]. High concentrations of ROS are associated with a decline in the cognitive functions reported in neurodegenerative disorders and neuroplasticity [20]. A large amount of evidence documented also that aging is characterized by a progressive deficiency of physiological functions due to the damage produced by free radicals to cellular macromolecules [21], and the strongest risk factors for the development of AD are represented by advanced age and carrying at least one apolipoprotein-E (Apo-E) ε4 allele [22]. Further, increased neuro-oxidative toxicity may explain, in part, the onset of MCI in the elderly [23].

Starting from these reflections, we wish to stress that that the brain and serological markers of OS should be study in-depth by future investigations in order to better describe the pathophysiology of AD, refine clinical diagnosis, establish a relationship with cognitive deterioration, and indicate future directions for research and therapeutics. The implementation of low-cost and well-tolerated probiotics in dietary interventions, healthy nutrition, physical exercise, and “being engaged in mental activities” representing critical lifestyles factors for mental vitality, may help in contrasting cognitive decline in the elderly, for which multidomain lifestyle-based prevention trials should be improved. In order to summarize the current evidence, we have recently contributed in reviewing the beneficial effects of antioxidants and brain/cognitive reserve to counteract the neurocognitive degeneration due to AD [24] by moving forward the potential onset during the course of pathological aging.
